# Venous thromboembolism in laparoscopic surgery: experience in elderly

**DOI:** 10.1186/1471-2482-13-S1-A8

**Published:** 2013-09-16

**Authors:** Rita Compagna, Gabriele Vigliotti, Tommaso Bianco, Maurizio Amato, Roberto Rossi, Francesca Fappiano, Giovanni Aprea, Bruno Amato

**Affiliations:** 1 Department of Clinical Medicine and Surgery, University of Naples Federico II, Via S. Pansini ,5 – 80131 Napoli, Italy

## Background

In the last years venous thromboembolism has became one of the most relevant social and health care problem due to: high incidence among patients who undergo surgery (20–30% after general surgical operations and 50–75% after orthopedic procedures), pulmonary embolism-related mortality rate, and long-term sequelae (postthrombotic syndrome and ulceration). This study has the purpose to establish how patient-related risk factors in elderly (over-70 years old) can modify the effect of laparoscopic cholecystectomy (LC) upon fibrinolysis and coagulation.

## Methods

This observational study included 90 low-risk elder patients for deep vein thrombosis (DVT) undergoing elective LC, without thromboprophylaxis. Preoperatively and after 24 h, we have evaluated following parameters: FDP, PT-INR, aPTT, d-dimer, fibrinogen. Color Doppler scan of the lower extremity has been performed during first post-operative day. Differences before and after surgery have been evaluated according to risk factors.

## Results

We did not appreciate clinically or ultrasound evident DVT. INR (1.04 ± 0.06 vs. 1.12 ± 0.11, p < 0.0001), d-dimer (0.38 ± 0.36 vs. 0.9 ± 0.64, p < 0.0001), plasma fibrinogen (380.8 ± 74.9 vs.403.8 ± 78.8, p ¼ 0.0001) and FDP positivity exhibited statistically significant increase after surgery. Levels of aPTT did not exhibit any significant change. Regarding d-dimer, elder age has been associated with higher pre-operative concentrations; elderly patients have exhibited higher increase in d-dimer and FDP after surgery. Male sex has been associated with higher PT - INR and aPTT before surgery, as well as with more pronounced increase in PT- INR during post-operative time; at same time elder age has been attributed with higher PT - INR before surgery.

## Conclusions

Despite no DVT, we have found significant increase in PT, INR, d-dimer, FDP and fibrinogen after laparoscopic surgery. These data could be the results of surgical trauma and pneumoperitoneum effects on the portal vein flow. Elderly subjects and males show major changes in terms of results.

## Competing interest

The authors declare that they have no competing interests.

## Authors’ contributions

BA: conception and design, interpretation of data, given final approval of the version to be published. RC, GV, TB, MA RR, FF: acquisition of data, drafting the manuscript, given final approval of the version to be published.

## Authors’ information

BA: Associate Professor of Surgery at University “Federico II” of Naples, Italy. RC: Post-graduate Doctorate in Vascular Surgery at University “Federico II” of Naples. GV: Resident in General Surgery Training Programme at University “Federico II” of Naples. TB: Resident in General Surgery Training Programme at University “Federico II” of Naples. MA: Resident in General Surgery Training Programme at University “Federico II” of Naples. RR: Resident in General Surgery Training Programme at University “Federico II” of Naples. FF: Resident in General Surgery Training Programme at University “Federico II” of Naples.
